# A Multi-Agent System Architecture for Sensor Networks

**DOI:** 10.3390/s91210244

**Published:** 2009-12-17

**Authors:** Rubén Fuentes-Fernández, María Guijarro, Gonzalo Pajares

**Affiliations:** 1 Departamento de Ingeniería del Software e Inteligencia Artificial, Facultad de Informática, Universidad Complutense, Madrid 28040, Spain; E-Mails: ruben@fdi.ucm.es (R.F.-F.); pajares@fdi.ucm.es (G.P.); 2 Ingeniería Técnica en Informática de Sistemas, Centro Superior de Estudios Felipe II, 28300 Aranjuez, Madrid, Spain

**Keywords:** sensor network, data integration, multi-agent system, metamodel, architecture, model-driven engineering

## Abstract

The design of the control systems for sensor networks presents important challenges. Besides the traditional problems about how to process the sensor data to obtain the target information, engineers need to consider additional aspects such as the heterogeneity and high number of sensors, and the flexibility of these networks regarding topologies and the sensors in them. Although there are partial approaches for resolving these issues, their integration relies on *ad hoc* solutions requiring important development efforts. In order to provide an effective approach for this integration, this paper proposes an architecture based on the multi-agent system paradigm with a clear separation of concerns. The architecture considers sensors as devices used by an upper layer of manager agents. These agents are able to communicate and negotiate services to achieve the required functionality. Activities are organized according to roles related with the different aspects to integrate, mainly sensor management, data processing, communication and adaptation to changes in the available devices and their capabilities. This organization largely isolates and decouples the data management from the changing network, while encouraging reuse of solutions. The use of the architecture is facilitated by a specific modelling language developed through metamodelling. A case study concerning a generic distributed system for fire fighting illustrates the approach and the comparison with related work.

## 1. Introduction

The growing availability of sensors pluggable into networks at low cost is rapidly increasing their use for different applications like smart spaces or surveillance systems [1]. These networks pose important challenges for engineers working in the development of the related control systems:

- *Potential high number of nodes*. The current trend is to set up networks densely populated with sensors and a minor number of controllers [2]. These magnitudes imply that engineers must consider issues such as the organization of the communications and local pre-processing of data to save bandwidth and achieve suitable response times.
- *Limited resources*. Sensors are devices with limited resources regarding memory, computational and communication capabilities, and, when they depend on batteries, energy [1]. This makes saving resources a key concern in the control systems of these networks.
- *Sensor heterogeneity*. These networks include a wide variety of devices (e.g., cameras, motion sensors and microphones) whose management and usage differs [3]. These sensors are usually specialized in specific applications, so they do not offer the same services. The combination of different types of sensors in a network and the use of their data require a high modularity and adaptability in the control system.
- *Unreliable networks of changing topology*. Sensor networks are less stable than traditional computer networks [2] because their components are more prone to failure. These components frequently operate unattended in environments that can lead them to malfunction, and they can exhaust their limited resources. A common way to overcome sensor failure is deploying new sensors, which further changes the network topology. These dynamic changes make that the control of the network must deal with *ad hoc* topologies to attend the communication needs of a given moment with the available resources.
- *Several levels of data processing*. Processing of data happens at both local and global levels [1]. Sensors are deployed over quite wide areas and external commands or computational capabilities are not always available. Thus, the sensors need to be able to deal autonomously, at least during certain periods of time, with aspects such as energy management or data collection pre-processing, and storage. Besides, the different locations of sensors may make necessary data contextualization, for instance to determine what signals are relevant. Nevertheless, centralized processing is also needed, mainly for the transformation and integration of data.

These challenges have been addressed in several works, though usually focusing only on partial solutions for some specific issues. For instance, [1,2,4,5] report work on routing in *ad hoc* networks to minimize energy consumption, optimal data processing to reduce computation time in sensors or data integration in specific domains. However, the integration of the different solutions is not a trivial problem and research in architectures for these networks pays attention to it.

The architectures proposed for these networks consider some infrastructure and/or a component model. The infrastructure provides basic services for all the components, and the component model specifies the interfaces and behaviour that components must provide to be integrated in the network. Nevertheless, these solutions also present some limitations. First, they usually require that systems conform strictly to certain features, such as specific interfaces or rules of behaviour (e.g., [6,7]). Second, most of these solutions does not cover the whole design and provide little support to address those aspects outside their focus (e.g., [3,8]). So engineers need to devote a relevant effort to work them out from available basic primitives. Third, there are not specific development processes linked to these architectures that facilitate their use indicating steps to follow, information to gather or good practices. Engineers completely depend on their own knowledge and skills to achieve successful developments. These features limit the usability of architectures and designs, which are only applicable in very limited contexts and by experts.

In order to address these limitations, some works [9] have proposed multi-agent systems (MAS) [10] as the basis for the development of sensor networks. A MAS is composed of a large number of agents and other computational artefacts. These agents are goal-oriented components, *i.e.*, they are modelled as entities that pursue goals and choose for execution those actions that will potentially contribute to satisfy them. These choices depend on their information about the environment, past experiences and themselves. Agents are also social because they need to interact with other agents to achieve the satisfaction of goals, and these interactions are modelled in terms of information, requests and informs. The works in this approach see sensors as devices controlled by agents [11,12]. In this way, sensors are only responsible of data gathering and basic processing, while complex and computationally expensive processes are assigned to agents. This approach to design offers several advantages. MAS [9] are well-suited to describe the interactions between components (e.g., information exchanged, related tasks or reasons for that interaction) and the way in which the network adapts to changes in it (e.g., new deployments or sensor failures) or its environment (e.g., low visibility or rising temperature). Besides, decoupling controller agents and sensors gives freedom of choice to locate agents with the data processing either mainly in the sensors (as proposed in [1]) or in the controller devices (as proposed in [7]). Despite of these common features, there are relevant differences between approaches (e.g., features of agents and sensors, focus or support for development), but none of them achieves a complete architecture and development process to design sensor networks.

This work addresses these issues with a solution that includes a standard architecture for sensor networks able to deal with different choices in design; a modelling language oriented to the kind of abstractions in that architecture; a development process for such systems. The language provides high-level abstractions to describe agents and sensors in the networks and how they interact. The architecture offers an extensible set of patterns to organize these abstractions. These patterns are partial reusable software design models. The patterns available at the moment regard dynamic adaptation to components, their features, and ways of processing data. The development process indicates how to refine these design models to the final code following a model-driven engineering (MDE) approach [13]. MDE proposes the generation of systems from their models through successive refinements of these models until the final code using semi-automated transformations. In this context, modelling languages are usually defined with metamodels. A metamodel indicates the kinds of entities, relationships and attributes, and the constraints of a modelling language. Transformations are as far as possible specified with standardized transformation languages for their automated execution. If that is not possible, engineers manually modify the models or code. To provide these elements, this work adopts as its basis a well-known model-driven agent-oriented methodology, INGENIAS [14].

The architecture considers sensor networks composed by devices (which include sensors) and actors (*i.e.*, agents). It extends common definitions for these concepts [11,12,15] in several ways. A device is defined as an environment element with attached functioning parameters, an internal state, and methods to work on that state. The device is also able to raise events in order to notify changes in its state. A sensor is a device that perceives events coming from its environment.

Actors are similar to controllers in other approaches, but the architecture introduces for them a neat separation of concerns with roles. A role is defined by its goals, which are related with its responsibilities, and the capabilities (*i.e.*, tasks) and resources (*i.e.*, devices and applications) it has to achieve them. Different role types have exclusive skills. For instance, only *device controllers* can communicate with devices, and the *group leaders* have the power to impose certain goals to the members of their groups. The current version of the architecture includes several predefined role types, but this list can be extended to address new needs of sensor networks, such as secure communications or resource assignment [1]. These roles are played by actors, which are agents with common inherited capabilities about goal management and task execution. Their specification focuses on how they implement the specific tasks related with their roles. The architecture defines teams of roles and their interactions to perform certain tasks, for instance, the setup or the dynamic addition of sensors.

The previous definitions of sensor, role and actor partially match those of INGENIAS external application, role and agent respectively. Nevertheless there are relevant differences. For instance, INGENIAS external applications and sensors are both environment elements characterized in terms of the methods they offer and the events they produce, but sensors extend applications considering their internal state, and how this changes as a consequence of external events and method execution. Thus, this research has modified the INGENIAS modelling language to accommodate new concepts. This modification enables applying its model-driven process for the development of sensor networks in our approach, reducing the related costs and error rates by raising the level of abstraction of development. For instance, generating the code for a target platform like nesC [16] and TinyOS [3] from the design models of the network requires a minimal engineers' intervention when the related automated transformation are available. This reduces the probability of coding mistakes. If the transformations are not available, engineers need to develop them, but they can reuse them in other projects for that target platform. This reduces the development time, and therefore costs, for the subsequent projects.

The approach presented is intended to be valid for different sensor networks. This paper focuses on the presentation of the general design aspects of the approach, so details about specific implementations are out of its scope. Nonetheless, the way to consider certain issues, such as limits on energy, memory or bandwidth, or addressing the development for a given platform, are briefly discussed regarding the development process.

The paper illustrates the approach with a case study about simulation for a cooperative architecture for fire fighting. The system includes two types of vehicles, Unmanned Aerial Vehicle (UAV) and Intervention Vehicle (IV), and static sensors. An UAV incorporates sensors to monitor the terrain, and processors to analyze their data. An IV includes ground sensors and maps to plan a route from its current position to the place where its intervention is required. There are also sensors in observation towers, mainly thermal cameras. The selected route depends on the real-time information provided by UAV and sensors deployed in the area. The main issues in this design are extensibility and scalability regarding types of vehicles, sensors and data processes, and the individual elements of these types. Consider that given the hostility of the environment and the potential long time of the intervention, such systems are probable to experience losses in the deployed units and sensors, as well as their capabilities. So, new elements may need to be added to the ongoing effort in order to guarantee a successful operation. This case study is also the basis for comparison with related work.

The rest of the paper discusses the elements in this introduction with further details. The first sections make a brief introduction to the foundations of our approach: Section 2 discusses MDE; Section 3 presents the INGENIAS methodology and modelling language. The next sections discuss the components of the presented approach: Section 4 introduces the extensions to the INGENIAS language to model sensor networks; Section 5 uses them to specify the architecture; and Section 6 describes the development process. The case study in Section 7 applies these elements to develop a generic distributed system for surveillance in fire fighting. Section 8 compares the results of the proposed approach with related work. Finally, Section 9 discusses some conclusions about the overall approach and the envisioned future work.

## 2. Model-Driven Engineering

Model Driven Engineering (MDE) [13] is a software development approach focused on models. Its perspective pursues rising the level of abstraction of development from code to models. This is expected to bring reduced development times and error rates, as less engineers' intervention is required to produce the software models of the target platform or the code, and to ease maintenance, as changing parts of the code of the network just requires modifying the models or applying different transformations. In this context, design models describe the information about the system and its environment. Automated transformations generate from them most of the different artefacts required to develop the control systems, such as new design models, documentation, code or configuration files. Engineers can develop such artefacts by hand, but it is largely recommended using models and transformations to keep traceability between artefacts. This kind of processing requires formal definitions of both models and transformations.

Metamodels [17] are a popular mechanism in MDE for defining the abstract syntax of modelling languages, that is, the primitives (*i.e.*, entities, relationships and attributes), constraints and rules needed for creating design models in that language. A metamodel is specified as a model written in a meta-modelling language such as ECore [18] or MOF [17]. One of the key advantages of using metamodels is the ability to extend or modify a given modelling language definition to address specific needs of a domain. There are several alternatives to make these extensions [19]. The most expressive, but also the more complex, is modifying the metamodel of the language. In the case of the Unified Modelling Language (UML) [20], the *de facto* standard for software modelling, profiles enable a simpler way of defining extensions. A profile identifies a subset of the UML metamodel or extends it with new standard or common elements, or well-formedness rules. Finally, it is also possible to use the inheritance mechanisms present in some languages to define new concepts. A concept that inherits from a so called super-concept has all the features of that super-concept besides its specific features, which additional models describe.

Transformations are the means to automate the life-cycle of artefacts in MDE [21]. There are three types of transformations: model, model-to-text and text-to-model transformations. A model transformation receives input from a model, conforming to a source metamodel, and it creates another model, conforming to a target metamodel. Model-to-text and text-to-model transformations work on models and text, such as documentation or code.

With these elements, a MDE project according to the OMG Model Driven Architecture (MDA) [22] is as follows. Engineers in collaboration with customers start with requirement models called Computation Independent Models (CIM). These are refined to abstract Platform Independent Models (PIM), which involve computational abstractions but are not tied to specific architectures or technologies. Then, engineers produce semi-automatically Platform Specific Models (PSM) from the PIM using model transformations with the help of Platform Models (PM). The PM contains the definition of the infrastructure for the sensor network (e.g., the operating system, programming language and libraries), and the PSM the design of a specific sensor network regarding that target platform. The PSM are used to generate the control system code with model-to-text transformations. Text-to-model transformations can be used, for instance, to extract models from legacy systems.

This kind of development encourages reusing of solutions, facilitates migration and reduces costs. Models and transformations represent knowledge about the solutions for given domains, and they are reusable between different projects. Thus, a given project usually needs only to develop from scratch some specific models and transformations, and reuse the rest of the elements from previous projects. Moreover, this approach facilitates platform migration. As transformations generate the final code, migrating to a different target platform just requires applying different transformations. Finally, the high degree of reusability of models and transformations, and working at a higher-level of abstraction than that of code, are expected to reduce the costs of developing the control systems.

In the context of our approach, MDE offers a neat decoupling between the abstract architecture with MAS described in PIM, and the specific low-level details of sensor management in PSM and PM. This makes easier the addition/replacement of sensor types, as the MAS architecture remains and only the PSM and its related transformations need to be adapted.

## 3. Agent Development with INGENIAS

INGENIAS [14] is a MDE methodology for the development of MAS. It comprehends a specific modelling language, a software process and a support tool. Following MDE principles, it defines its design modelling language with a metamodel. This metamodel is the basis for the semi-automated development of its tool, and also guides the definition of the activities of its software process.

MAS in INGENIAS are organizations of agents, which are intentional and social entities. Agents use applications, which represent the environment and system facilities. The models to specify these MAS describe their environment, agents and interactions, both from the static and dynamic perspectives. The modelling language also includes a simple extension mechanism for agents through inheritance relationships: a new sub-agent type inherits all the features of its super-agent type, but it can also extend or constrain them. Table 1 shows the main INGENIAS concepts used by our approach.

The support tool of the methodology is the INGENIAS Development Kit (IDK). It provides a graphical environment for the specification of MAS design models. The tool can be extended with modules. The standard distribution includes modules for documentation and code generation based on templates. A template is a text file annotated with tags. These tags indicate the places where information from models has to be injected to get a proper instantiation. The instantiated template can describe, for instance, the code for an agent in a framework, the documentation of its goals, or the configuration files for its deployment. Engineers can use code components in models to attach specific code to entities. For instance, if engineers want to generate nesC [16] code, they first need to develop a template with the general description of an agent in that language; then the code generation module reads the design models of the sensor network, and for each agent appearing in them, it generates its specific code for nesC instantiating the template (*i.e.*, replacing the tags with information from the models that includes the code components).

There are two main reasons for the choice of INGENIAS in this work considering available alternatives with agents [9]. First, its modelling language is a suitable basis for the extensions required for sensor networks. It considers concepts such as agents, roles and environment applications that are required in our architecture, and covers the interactions between system components with a high-level of detail. Second, INGENIAS strictly adheres to MDE principles. It defines its modelling language with a metamodel that is also the basis of the IDK development. This facilitates the modification of the language to house additional concepts and propagating these changes to the tool. Given the complexity of the development of sensor networks [1,2], the availability of support tools (e.g., for coding, debugging or reporting) is mandatory to get a high productivity. Nevertheless, the IDK has the shortcoming of using an *ad hoc* approach for transformations based on modules and templates, although there are ongoing efforts to support more standard approaches [23]. The development process proposed in our work adopts standard transformation languages [21] to manipulate models and code. This has two key advantages. First, the tools to develop and run these transformations are already externally available, so there is no need of new developments. Second, these languages focus on the description of the transformations, which facilitates their understanding as this is not blurred with low-level details about processing design models (e.g., reading the input file, managing syntax errors or generating the output file).

## 4. An Agent-Based Modelling Language

**Figure 1.**
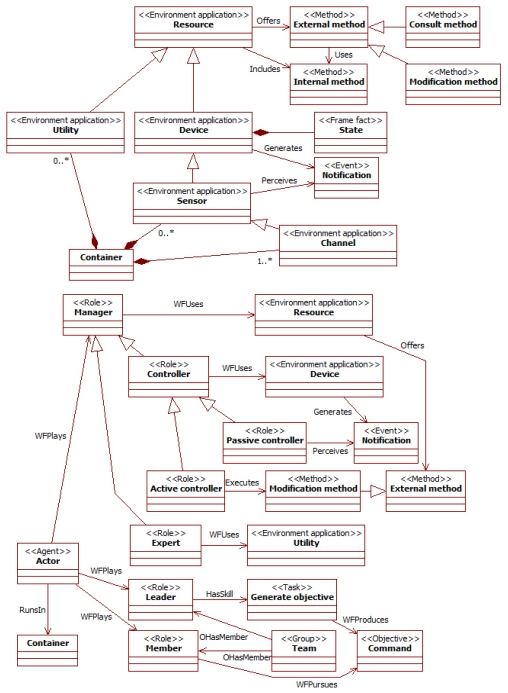
Partial metamodel of agent-based concepts for sensor networks.

The design of MAS to manage sensor networks in the presented approach uses specialization of general agent concepts. The purpose of these specializations is acting as a guide for engineers: They indicate the concepts that should appear in the specifications and how they are related. The main extensions of our approach to the INGENIAS [14] conceptual framework appear in Figure 1 with their main relationships. The mechanism used for the metamodel extension is its direct modification [19]. Note that profiles cannot be used since this is not an UML extension, and INGENIAS limits inheritance to agents (see Section 3).

Figure 1 represents elements of the metamodel of the modelling language in our approach. Nodes and links respectively represent meta-entities and meta-relationships. Meta-relationships with triangles and diamonds are standard (*i.e.*, non specific of INGENIAS) representations of inheritance and aggregation (*i.e.*, whole-part link) relationships. Numbers in the ends of relationships are cardinality indications. The stereotypes of nodes (represented between angle brackets) are the names of the INGENIAS meta-entities that our meta-entities extend. The meta-entities have the features (e.g., attributes and relationships) of the extended meta-entities and add new features and constraints. For instance, at the meta-modelling level, there are meta-entities *device* and *controller* that are modifications of the INGENIAS meta-entities *environment application* and *role* respectively. These meta-entities are related with a meta-relationship *WFUses*, which is restricted to connect this pair of meta-entities. These meta-elements are instantiated in models. For example, a model can contain instances of the *device* meta-entity, which can only be related with instances of the *controller* meta-entity through instances of the *WFUses* meta-relationship. The rest of the section discusses the concepts present in Figure 1. Some examples of their use can be found in Section 5.

A sensor network in the proposed architecture distinguishes between reactive and active components. Reactive components receive requests or notifications of events, and generate answers for them that only depend on the input and some internal state if this exists. Active components take initiatives on their own that contribute to satisfy the system goals. The basic type of reactive component is the *resource*, and the *actor* of active component. Actors are a specific type of agents that use resources. Their work is organized through the roles they played. Roles represent prototypical aspects of the activities in the network. A role indicates the goals it pursues and the available elements to achieve them, which can be information, capabilities and resources.

A *resource* is an external application. Its specification is known, but neither its behaviour nor its interfaces can be modified. The only way to interact with it is what their external/public interfaces allow. The actions available for this external manipulation of resources are represented by *external methods*. These methods can change the internal state of the resource, *i.e.*, *modification method*, or just consult it, *i.e.*, *consult method. Internal methods* can be used to provide information about the internal behaviour of the resource with specification purposes, but other components of the MAS cannot invoke them. A resource may have functioning parameters that influence its behaviour. These parameters can determine for instance, the threshold of certain operations or the initially available energy. Resources represent different elements appearing in sensor networks. A *utility* is a stateless resource. It corresponds to a computational facility available for the network, such as data normalization, combination of different signals or information conversions. *Devices* are stateful resources able to generate events called *notifications*. The state is characterized in terms of frame facts, which are the units of information in INGENIAS. Devices offer specific methods to manage the subscription of other components to their notifications. A subclass of devices is *sensors*, which generate events but are also able to perceive them in the surrounding environment. Thus, the behaviour of a sensor is characterized in terms of a state machine that changes its state according to the execution of methods and the appearance of events from the environment. A *channel* is a particular type of sensor intended for communication. It is able to send and receive information over a medium and perform basic tests on it.

These resources are used by *manager* roles to provide services in sensor networks. The language distinguishes two types of managers depending on if they work with devices or utilities. The *controller* is the role with access to devices. According to the rights it has over it, there are two types of controllers. A *passive controller* can only consult the device state with consult methods and perceive those events to which it subscribes. The *active controller* is able to make requests to change the device state using its modification methods. In this way, several access levels can be granted to controllers of the same devices.

The *expert* is the role in charge of utilities. This role specifies the knowledge and skills required to manipulate an utility, as well as how to obtain additional information that can be extracted from sequences of data manipulations over time. For instance, an expert can store information about temporal series of signals to draw conclusions about trends.

Another concept central in the proposed solution is that of *team*. A *team* is a hierarchical INGENIAS group that comprehends a *leader* role and several *member* roles. The leader has the right of posing new commands to the *members* of its team, where a *command* is a kind of objective. Roles receiving the commands must include them in their agenda, but their management of them depends on their design. The leader can be also the provider of a given service for all the members of its team. Teams facilitate setting up basic groups of collaborating roles. For instance, there are groups for the initialization of the network, solving issues of quality of service, communications or data processing. These teams constitute design blocks that can be reused in different specifications.

The previous roles are played by roles and actors. When a role plays another role, it adds the features of that role to its own ones. The actors are agents with common skills for the management of goals (e.g., decomposition, checking their state or removing when satisfied), planning for their achievement (in terms of the available information, resources and capabilities) and basic communications (both with agents and resources). When an actor plays a role, it fulfils the standard behaviours specified by the role, that is, it implements its capabilities, has actual access to its resources, and manipulates the related goals and information. The actor can have additional elements beyond those of its roles. Note that an actor manipulates all these elements globally. For instance, the satisfaction of a goal linked to a certain role can be the result of the information produced with a capability related with another role.

The previous elements run in *containers*, which represent deployable computational devices. A container has basic processing capabilities that allow the execution of agents, and at least one channel for communication. Additionally, it can include an arbitrary number of resources. Note that, given the relationships and constraints in the metamodel, a device and its managers run in the same container.

In order to provide a simple extension mechanism for the language, this approach also generalizes the INGENIAS inheritance relationship. It is not constrained to agents but can be applied to any concept with an equivalent meaning: a sub-concept inheriting from a super-concept has all its features but it can extend or constrain them with additional models.

## 5. Architecture for Sensor Networks

The metamodel for sensor networks just defines the modelling primitives that can be used when specifying these networks as MAS. However, it cannot specify how these elements should interact to provide the expected functionality. The architecture provides this information. This section focuses on its description through its main teams. The list is not exhaustive, as more teams can be specified to address new needs. The description of teams includes their purpose, and the characterization of their leader and member roles regarding their responsibilities. Note that when talking about roles performing actions, it is really the actors playing those roles that perform the actions, as roles are just functional abstractions.

The *initialization* team is aimed at setting up all the components of a container and providing them with the initial information required for their proper functioning. Its team leader is the *initializer* and its members play the role of *targets* of the initialization. The initializer creates all the actors in its container and sends them the information about the managers they play. Then, each manager receives a list of the assigned resources, and the notifications and external methods it can use. If required, it can also obtain information to initialize the resources. Besides, each manager receives information about all the teams it belongs to, including the type of team, its leader and the role of that manager in it. Note that these teams can involve roles whose actors are not running in the same container.

An *information process* team focuses on the generation of information from the data of devices. Its team leader is a *consumer* for that information. It organizes the gathering and processing of data. Team members play the role of *providers* of information and can be passive controllers or experts. The activity of these teams can begin either with a request from the customer or with a notification from a device. In the second case, a passive controller provider captures an event raised by a device and notifies it to its consumer. From this point forward, both scenarios are the same. The consumer may send additional requests to its manager providers: to passive controllers in order to collect additional data; to experts to further manipulate these data before their use. Note that with this approach, the consumer itself can be regarded as a manager that provides services of a higher-level, as it encapsulates the interactions with a group of resources and its managers.

*Communication* teams refine the INGENIAS communication schema, as they give further details about how interactions are transmitted between different actors and roles. They manage communication through *channels*. The *communicator* is both the team leader and the active controller of the channel. The rest of the members of the team play the role *customers* in the communication. All the customers in a communication team are able of direct communication between them, but they need to ask its communicator for external communication outside the team. These teams encapsulate the use of the communication infrastructure and related algorithms, which makes transparent the communication capabilities of other network elements from the design point of view. Engineers only need to guarantee that each role or actor that needs to communicate belongs to a communication team in order to have access to a communicator. In order to optimize communications (e.g., latency or energy consumption) and perform message routing, communicators need to build a rough map of the nearby communicators. Containers have a limited range of communication, so some messages may need several hops to reach its final destination. To build the map, a communicator broadcasts a request of information amongst other communicators in range. Available communicators answer this request with information about the features of their service, and take note of the sending communicator.

*Load balancing* teams are intended to keep the quality of service in the network. Sensor networks face to several situations that can require their dynamic reconfiguration. Some of them were outlined in the introduction, such as failure of sensors or communications, but also sensors overloaded with requests or replacement of the failing customer for some data. Although different, all these situations are solved through the collaboration of two sub-teams. First, there is a *failure notification* team where a team leader *referee* controls a group of team member *watchers* that can warn of potential failures in the behaviour of some observed elements of the network. For instance, a controller can be the watcher of a sensor: when this sensor depletes its energy, it does not longer answer the requests of its watcher, which raises to its referee the information about the failure. The referee evaluates that information and if it determines that there is need of acting, a repairer team begins working. A *repairer* team has as leader a *dispatcher* governing a set of *referees* and *initializers*. When a dispatcher receives the notification of a failure, it looks for some replacement. The replacement can be obtained either asking other referees in the team for a component with similar features or asking an initializer to create a new one if possible. For instance, in the case of failure of a sensor, the replacement could be another sensor in a container near the location of the original one, but if an expert is failing, a new one can be created and assigned to the utilities of the original one. The dispatcher informs of the replacement to the involved referees, which send to the initializers in their containers the information to update. For instance, adjustments need to be made in the state of the replacement or the teams depending on it.

Note that any container must have running at least two teams. The initial setup requires one initialization team, and integration with other elements of the network a communication team. Executing these teams requires at least one actor which plays the initializer and communicator roles.

The architecture involving these teams pursues satisfying three main objectives. First, it facilitates the design of sensor networks decoupling the different responsibilities in roles and teams. Second, it looks for networks that can semi-autonomously reconfigure themselves to address new situations, a concept present in current research in autonomic computing [24]. Third, it achieves the extensibility of the design of systems to control sensor networks through new teams. An example of these extensions can be seen in the case study, where the control is modified to deal with new types of sensors.

## 6. Development Process

**Figure 2.**
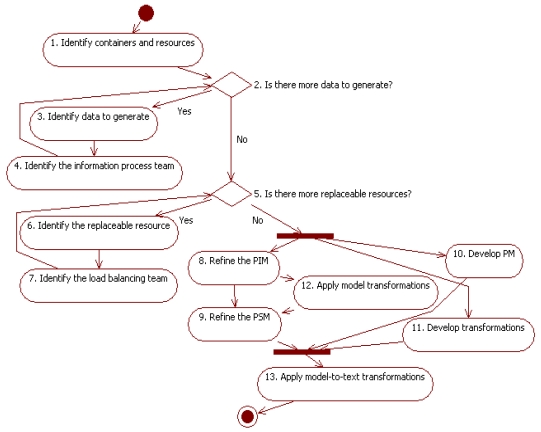
Process for the development of sensor networks.

This work includes a simple model-driven development process customized to develop the control system of sensor networks following the architecture in Section 5. As explained in Section 2, a model-driven process focuses the development on design models. Engineers refine these models from abstract representations to those models closer to the intended target platform, and finally to code. The refinements are partially supported by automated transformations. The process proposed in this approach is based on the software process of the INGENIAS methodology [14]. It adds to the INGENIAS process several specific activities aimed at identifying the elements required in a sensor network. These elements are those defined in the modelling language (see Section 4) and organized in the teams of the architecture (see Section 5). Figure 2 shows the resulting process. Activities 1–7 are specific of the current approach, while Activities 8–13 summarize INGENIAS activities. The process takes as input a previous analysis of the data required as output of the network and the sensors able to provide them, and produces as output the code of the control system for the sensor network.

The design of the network begins with Activity 1. Engineers determine the containers of the network, *i.e.*, the computational devices able to execute code and transmit information. These are usually the sensors, but also additional devices such as computers or communication facilities can be considered here. This activity also identifies the resources: the sensors that gather data from the environment; the utilities that represent services that actors use to process data. The activity distinguishes the two aspects of the sensor, as resource and container. Note that the modelling language provides different concepts for these aspects, and therefore assign to them particular features that must be considered in the design models. When these elements have been identified, engineers assign them initialization and communication teams. As discussed in Section 5, these teams are mandatory for every container.

Decision 2 and Activities 3–4 are intended to organize complex processing and integration of data. According to the architecture, information process teams are responsible of these activities. Engineers identify in Decision 2 and Activity 3 specific data that must be generated in the network. For each group of data, Activity 4 designs the corresponding team. First, engineers discover the sensors that provide the source data. For each sensor, they must assign at least one active controller and a passive one. The first one is required in the initialization, and the second one to provide access to sensor data. Next, engineers must identify the data transformations required to get the final information. Some of them are achieved using utilities of the network. For these utilities, engineers assign an expert. Finally, the team is composed by the passive controllers of the sensors and the experts of the utilities playing the role of providers, and a customer to integrate and consume the information. The identification of this kind of teams finishes when all the complex calculation of data has a team assigned.

Decision 5 and Activities 6–7 are intended to specify the teams that manage the dynamic adaptation of the network. Engineers begin this design with Decision 5, where they find out what the elements are that can fail or be incrementally set up or deployed during the working of the network. This identification considers resources, roles and actors. For every element identified in this decision, Activity 6 carries out an analysis regarding its potential replacements, and how they can be located and evaluated to find the best suited if several are available. Activity 7 designs the specific team related with this replacement. It includes a watcher that monitors the element. In the case of a device it is a passive controller, for a utility it is an expert, and for a role or agent it can be a customer that communicates with it. The team also needs a referee that evaluates when the failure needs to be notified for a potential replacement. The repairer team includes referees related with the same type of elements and similar features. For instance, for sensors they can be referees of nearby sensors and for roles other agents in the same container able to work with the same resources. As an alternative, initializers can be used to set up new roles or agents in these teams. Each of these teams must also identify its dispatcher, which selects the best alternative for a required replacement.

After these activities, engineers have available PIM of the resulting system according to the architecture. These PIM describe the devices, agents and roles, the information they exchange, and their interactions; they do not contain details on the final target platform, for instance about energy levels or low-level control commands for the sensors. Activities 8–13 follow the INGENIAS process to refine these models and generate the final code of the control system.

Activity 8 adds several INGENIAS PIM to the MAS specifications. Organization models define agents and groups outside the architecture, and assign to the groups workflows that describe their work. This allows refining the teams when complex processing of data needs further specification. Agent models refine actors and roles with additional goals, capabilities and information. These models also establish the pieces of information whose appearance determine when a goal is satisfied or failed. Tasks/Goals models map tasks with the goals that satisfy them, and hierarchically decompose goals and tasks into sub-elements. Interaction models describe actor interactions in terms of goals pursued, information exchanged and tasks performed. These models provide the details of the previous architectural design, though they are not always required. For instance, if engineers do not need to refine teams beyond what is said in the architecture, they do not use organization models.

Activities 9 and 10 develop the models required for the final target platform. Activity 10 develops the PM corresponding to the target platform. These PM include information about how to translate general concepts to specific elements in the platform. As explained in Section 3, INGENIAS uses templates to represents PM. For instance, in the case of the Matlab (http://www.mathworks.com) implementation of a sensor network in the case study, it indicates the structures used to implement types of resources, their methods and their events. In case that these PM are available from previous projects, Activity 10 can be omitted. Activity 9 develops the PSM of a specific design for the target platform. The PSM provide two main types of information. First, resources include their functioning parameters for the target platform, which can describe their limits about energy, memory or computational power. Second, engineers provide with code components attached to modelling entities the code specific for them. That is, part of the code required for the final system cannot be extracted from models, as models abstract the specific low-level details of the behaviour of systems. For instance, there are not modelling primitives to describe complex algorithms, and templates only contain general code for concept types in a platform. Engineers can include the remaining information attaching INGENIAS code components to the elements in models.

Activity 11 considers the development of the transformations that support the semi-automated refinement of PIM to PSM in Activity 12, and the generation of code from PSM in Activity 13. In the case of an INGENIAS development, transformations are implemented as IDK modules. These modules support model transformations and model-to-text transformations. Model transformations are useful to represents standard refinements of model concepts. For instance, each actor needs several goals to manage its planning cycles (e.g., collect information, discard non-achievable goals, look for achievable goals), but these are standard and engineers do not need to write them for each actor; a transformation can automatically generate these goals for the available actors. The best-known example of model-to-text transformation is code generation. In this case, the IDK includes a module for this purpose in its standard distribution. For a given specification and target platform, this module operates as follows: (1) it identifies the templates for the concepts present in the specifications and the target platform; (2) it traverses the templates looking for their tags; (3) when it found a tag, it replaces the tag with information from the models, which can be the content of a code template; (4) it returns the instantiated template as its output, which is the code of the concepts. In this way, changing the target platform for a given design only requires using different PM (*i.e.*, code templates) and changing the attached code components. For instance, changing the target platform in the case study from Matlab to nesC does not need changing the design, it only needs using the nesC templates, modifying the code components of entities, and running the code generation module of the IDK to produce the code.

Note that though Figure 2 shows a sequence of activities, a true development needs to carry out several iterations of these activities. For instance, engineers can discover when they are developing their PSM in Activity 9 that some teams are missed, and they will need to return to Activities 2–7. Moreover, Activities 1–7 need further refinement to provide more guidance depending on specific application contexts.

## 7. A Case Study: Disaster Intervention

Disaster intervention [25] is an illustrative application of sensor networks challenges and opportunities. It implies the use of large numbers of sensors of different types in a hostile environment, over a non-determined period of time. Among the different scenarios, this case study focuses on fire fighting in the countryside [26]. One of the main concerns in these situations is having available accurate and updated information about the terrain and the evolution of the fire. Teams usually rely here on their good knowledge of the place, and manned aerial support to monitor in real time the evolution of the situation. However, the conditions of the disaster, such as an abrupt environment, thick smoke, strong winds or an area too wide for the available resources, can worsen the situation. In this case, manned aerial support may not be available with a proper coverage for all the teams. To deal with this kind of situation, researchers [26,27] have proposed the use of sensor networks. Examples of sensors available in this case can be thermal cameras, humidity sensors, smoke detectors or microphones. These sensors can be deployed in fixed locations like observation towers or over the terrain [26], or can be mounted on mobile platforms, for instance, Unmanned Aerial Vehicles (UAV) [27]. The advantages of using sensors in these situations are that they can act in places and under conditions where human access is not possible or too dangerous, and large numbers of them can act at the same time tracking and helping localized intervention teams.

**Figure 3.**
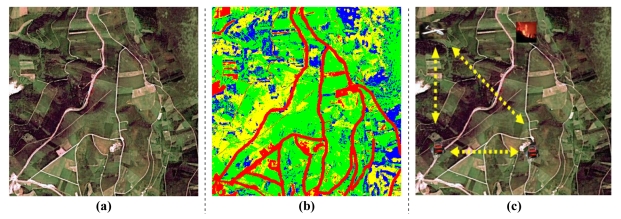
The fire fighting disaster intervention. (a) Picture taken from the UAV camera in optimal visibility conditions. (b) Semi-processed image with textures. (c) Path planning in the IV.

The design proposed in this case study combines both fixed and mobile sensors in an incremental way, showing the scalability and adaptability of the approach to changes in the configuration of the network. It considers a set of Intervention Vehicles (IV) with fire-fighters. The crews of these IV need to arrive to the place of their intervention from their current positions following the safest practicable routes. For this purpose, the system in the IV that computes the path has available maps of the zone and integrates the data from sensors. In the first stage of the design, the available sensors are only image cameras in UAV. The second stage integrates in the design static sensors located in observation towers. The resulting system works as follows. The sensors capture information (see Figure 3a), and partially process it (see Figure 3b). They send the semi-processed data to the IV, which integrates and further process them (see Figure 3c where sensors in UAV communicate with the IV). The design of the sensor network for this setting must pay special attention to flexibility about deployed components and their types, and their reconfiguration over time to keep the service within the safety parameters for the fire-fighters.

The development of the proposed system has followed the process in Section 6. A first simulated prototype has been developed under Matlab. In this prototype, the data that should be provided by the sensors are loaded from a database, but not acquired from physical sensors. Sensors are simulated by software components wrapping the interaction with that database. The actors of the network receive and process these data as for the actual system.

The first stage of the design of the system (*i.e.*, with IV and UAV) begins with Activity 1, which identifies the available containers. A container (see Section 4) must provide at least hardware to run actors and a communication channel, and may include sensors. In this case, there is a neat division in containers: one for each UAV and IV. Both types of vehicles are equipped with computational devices able to run some software, and in particular the actors of the architecture, and that have available a channel for radio communication. These containers also include some sensors. First, each vehicle has to provide accurate information about its position. For this purpose, they carry Global Positioning System (GPS) devices. Second, the actual state of the terrain is obtained from cameras in the UAV. As explained in Section 5, every container requires at least initialization and communication teams. The information delivered during the initialization includes the data about the teams described below in this section.

Decision 2 and Activities 3–4 identify the information process teams. The first activity specifies data that must be generated and the second activity designs the corresponding teams. In this case, there is one information team that consumes the data of the GPS and cameras. It includes passive controllers for these sensors as providers. The consumer is in the IV. It finds the UAV acting near its IV with the information from the GPS. From these UAV, it gets the data of their cameras. It uses the services of a provider expert with access to a utility for image and map integration to get the actual map of the terrain. Depending on the processing required on these data, the system may also need additional experts. For instance, images have to be processed to determine the textures in them and find the zones and structures of interest (see Figure 3b). The containers for the experts depend on where their utilities are installed. Since IV have a higher load capability, and hence they can carry more powerful computational devices, these actors run in their containers.

**Figure 4.**
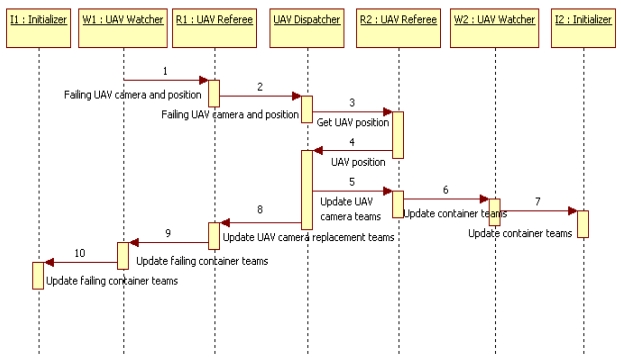
Replacement of an UAV camera serving an IV.

The system may also require load balancing teams. The engineers identify these needs in Decision 5 and Activity 6. This system faces the potential loss of sensors in UAV during the intervention, as they can break down or their UAV need to return to its base. As IV cannot be left unattended, the lost sensors have to be replaced. Activity 7 carries out the design of the related load balancing teams. The corresponding failure notification teams monitor UAV cameras. Both the referee and watchers of these teams are in the IV using the camera data. Repairer teams include the previous referees and dispatchers for cameras. When the dispatcher receives a request for the replacement of a UAV camera, it asks to its referees about the current location of their related UAV. The dispatcher matches the GPS information of these UAV with the location in the request to decide the best replacement and informs about it to the original referee. Note that this workflow requires that actors playing the roles of watchers and referees also play the role of consumers in process information teams for GPS data. The sequence, excluded collection of data from sensors, can be seen in Figure 4. The original watcher W1 finds a failing UAV camera and informs to its R1 referee in the failure notification team. R1 evaluates this failure and approves it for its dispatcher in the repairer team. The dispatcher looks for the replacement with other referees R2. If the dispatcher finds a suitable replacement, it asks the involved referees R1 and R2 to modify the data related with that UAV camera. This information concerns teams and containers where the members of those teams run. For instance, consumers of information must refer to the controllers of the new camera instead of to those of the broken one.

Turning back to the initialization team and according to this design, the following information needs to be provided. First of all, engineers initialize the target areas where the vehicles have to work. In a simplified setting, people would lead vehicles to their areas. After that, initializers have to do the initialization of their containers. They set up the actors that are going to play the roles. For the GPS and camera sensors, they create the active and passive controllers, and feed up the active controllers with the information to initialize these sensors. The last information they have to provide to actors is about the information process and load balancing teams previously described. Engineers indicate to each actor receiving the information the roles it plays and the leader actors of the teams if it is not.

**Figure 5.**
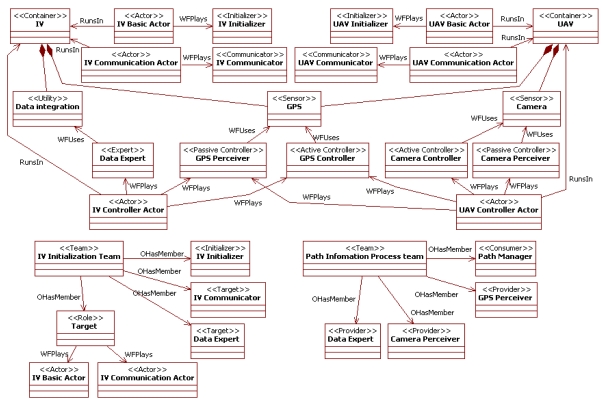
Partial design of the system after the first stage. It includes the containers and their elements, the initialization teams, and the information process team.

Some of the elements of the design identified after the first stage appear in Figure 5. The diagram considers the system with only IV and sensors in UAV. It includes the containers and their elements, the initialization teams, and the information process team. Stereotypes (between angle brackets) are the names of the meta-entities from the modelling language (see Section 4) and the architecture (see Section 5). The names of the entities belong to the design of the actual sensor network.

After establishing the teams, the rest of the development follows the standard INGENIAS process [14] in Activities 8–13. As it is a model-driven process, it is focused on the generation and refinement of design models and their related transformations.

Engineers refine the MAS design models with Activity 8. They describe for each role and actor the decomposition of its goals and tasks. This activity also details the information of the system (*i.e.*, the frame facts of INGENIAS) and uses it to set up workflows. These workflows indicate which tasks are performed by each agent/role, attending to which objectives, and the pieces of information that those executions require and produce. Note that a task can be related with different goals and pieces of information depending on the context. Tasks are also used to manage the execution of methods of resources. Activity 9 performs the specification of the PSM. At this stage, functioning parameters (e.g., energy, memory or communication range) can be assigned to sensors if they were not previously. This activity also includes the fine grained specification of methods with code components (see Section 3), for instance to specify a control algorithm for a sensor that needs to deal with low power consumption or some data integration.

In this case, engineers do not need to perform Activity 10 to develop transformations. They manually refine the PIM to PSM, and the IDK already offers a module for the transformation of design models to code. As the refinement from PIM to PSM is manual, engineers also omit Activity 12. However, engineers need to develop the templates for Matlab that implement the concepts of the architecture in Activity 10. When models contain enough detail, engineers run the code generation module of the IDK in Activity 13. If further modifications of the control system are required, they change the PSM and automatically generate again the code.

In order to show the flexibility of the architecture, this case extends the control of the sensor network in two ways: first, to consider sensors in observation towers; second, to actively modify the location of UAV.

As said in the introduction to the case study, this kind of solution commonly combines mobile and fixed sensors. The design has only considered cameras in UAV, so let extend it with the fixed cameras. These cameras are going to provide additional information to the application for the computation of paths in the IV. As done for the UAV, the design needs to include a container for the new sensors with its initialization and communication teams. The design also needs active and passive controllers for these sensors. All these roles and the actors that implement them, reuse general solutions of design and implementation. Only the controllers need specific code to manage their sensors. The passive controllers become part as providers of the information teams previously identified. If new utilities are required for the integration of these data, engineers set up the corresponding experts and make them part of these teams. Finally, if required, these sensors can be integrated in load balancing teams.

The second issue considers that the previous solution is not completely satisfactory for the fire intervention problem. The initial deployment of UAV can assign several of them to the same area. Subsequent failures can make necessary changing the location of some of them to attend areas with less coverage. With the previous version of the system, the dispatchers cannot modify the position of the UAV, and therefore of their cameras. In order to being able to perform this kind of changes, the following modifications are introduced. The MAS includes new information process teams to govern the position of UAV. The consumer of this team has a path objective. Its providers are the controllers for the sensors of the flaps and motors of a UAV. These controllers can change the position and altitude of the UAV. In order to determine the current position of the UAV, this team also needs as providers the managers of the GPS. The second change affects the dispatcher of UAV cameras. Its capability to determine a suitable UAV for the request is modified as follows. Instead of answering with the nearest UAV to the IV, it looks for a UAV in an area with a high coverage. Then, the dispatcher notifies to the original referee (*i.e.*, the one asking for a UAV camera) the new UAV assigned to its area, and asks to the referee currently controlling that UAV to send it to the new location using the services of the new information process team. In this way, just considering the new UAV controllers for flaps and motors (which require specific implementation), a consumer to plan paths and a team that gathers these elements, the behaviour of the network has been modified without affecting other elements.

As a final remark, it should be noted that this approach is mainly focused on the design of the network. Specific implementation issues must be addressed with code present in the PM and PSM. Nevertheless, the fact of organizing development around design models and transformations facilitates a wider reuse of solutions among different developments, even for those solutions directly related with the low-level details of the network.

## 8. Related Work

This section compares the proposed approach with related work in sensor networks and MAS, considering both their general features and the requirements of the case study in Section 7. The introduction already discussed different perspectives on the design of sensor networks. This section follows this classification and distinguishes between integral solutions with architectures and partial solutions for specific aspects. Among architectures, there are examples focused on the infrastructure and others on the high-level design of the network. Transversal to these approaches, some researchers have proposed the use of MAS for the development of the related control systems.

Architectures for sensor networks focused on infrastructure provide a platform with basic services for the sensor network. This platform has a component model that those elements to integrate in the network must fulfil. In this group can be included operating systems (e.g., TinyOS [3] and Contiki [28]), programming languages (e.g., nesC [16]) and middleware (e.g., MORE [8], RUNES [29], SMEPP [6] and Tenet [7]). These works and ours appear at different levels of abstraction when considering the development of sensor networks and their control systems. According to MDA [22], the models based on the architecture proposed in this paper are PIM that use highly abstract primitives to model sensor networks. These abstract elements are mapped to the constructions available in these implementation platforms. For instance, the concept of team in our architecture can be partially supported in SMEPP [6] with the concept of group, which provides mechanisms for authentication and authorization, communication between agents can be implemented with μSOA messages of MORE [8] or as said before the case study can replace its implementation in Matlab with another in nesC [16]. The information of these platforms would appear in our approach as PM. Engineers would refine the PIM of our architecture in that provides the information for that specific implementation. This refinement would be partly implemented with automated transformations from PIM to PSM, for instance to create the structure of μSOA messages, and partly manual, for instance the actual content of messages. If required, abstract components of these architectures could appear in the architecture of this paper as additional roles and teams. The code generation module of the IDK would generate the code for the control system from the final PSM and PM.

Architectures considering the high-level design of the network have adopted usually the form of guidelines. Either they just give some abstract design principles (the case of [7,11,30]) or they consider also a development process (the case of [12,15,31]). From the point of view of the design principles, the flexibility of the proposed architecture allows it adopting the principles underlying a variety of these approaches. For instance, carrying out the processing of data as close as possible to their sources (as [30] recommends) means that the actors playing the roles of information process teams should run in the same container, and moving that processing to more powerful computational devices (as proposed in [7]) splits these actors in different containers. In both cases the design of roles and teams is the same, and only the initialization information actually changes. The proposed architecture is not intended however for mobile agents as those in [30,32]. Actors in the proposed architecture are not able of redeploying in a container different from that where the initializers create them. However, the initializers could be modified to allow this kind of behaviour. It would be enough to allow initializers to collect information about the actor that wants to migrate (e.g., current state, teams or available resources), and send it to the target container where another initializer would use it to create another actor with the same data. Of course, this migration would also demand checking that the resources and managers that the actor needs are available in the target container.

This section has already mentioned works based on agents [11,12,15,30,31], but some of them deserve further discussion given the similarities with our work. [11] establishes some guides for the design of MAS for sensor networks and uses some concepts common with our approach, as controllers, sensors and providers. They also consider concepts that our approach can incorporate, such as directory facilitators to refine the location of sensors with certain features. However, these roles are informally defined in terms of their responsibilities and the set is closed. In this sense, our approach with a specific modelling language and the possibility of defining teams facilitates customization. Besides, [11] does not consider a development process for control systems. [12,15,31] present development process for control systems. ARTIS [15] is a methodology for holonic manufacturing systems that includes the use of sensors. It considers aspects of real time, but ignores issues such as limited resources. [12] is tailored for sensor networks and has been validated with real projects. Though it considers automated generation of code, it does not offer a standard process for it, as our approach does with MDE. This makes more difficult reusing available infrastructure for development and reusing the design models of previous projects. [31] deserves special mention as it also considers INGENIAS for the design of sensor networks. As a matter of fact, both approaches represent complementary points of view. The approach proposed in this paper extends the modelling language of INGENIAS with new concepts, and establishes patterns and guidelines to address the design of these networks with its architecture. These tasks correspond to Activities 1–7 in Figure 2. Since these models are INGENIAS models, their refinement to the running code can follow any suitable INGENIAS development process. These tasks correspond to Activities 8–13 in Figure 2. In particular, this refinement can follow [31], which is targeted for sensor networks. Thus, these works can be seen as part of an ongoing effort to provide engineers with a tailored methodology and development process for sensor networks.

Finally, a last group of approaches (mentioned for instance in the surveys [1,2,4,5]) considers specific solutions for aspects of sensor networks. Depending on the issue that they address, they are integrated in different elements of the proposed architecture. Algorithms for routing in *ad hoc* networks are part of the responsibilities of the communicator role in communication teams. Choosing the best place to process some data in order to minimize energy consumption is a design decision about the information process teams. It determines the containers of the consumers and the providers. Issues about data integration are addressed as part of the specification of utilities, their controllers and consumers of information also in information process teams.

The case study in Section 7 focuses on the design of the system and has not discussed issues such as power consumption, specific data integration problems or routing. Research about these aspects can be integrated in the solution for this problem as previously mentioned in this section. Concerning the design, the case study shows two relevant limitations of existing approaches. First, the scope of some of the approaches is not well-suited for the presented problem. Approaches as [3,8,29] do not facilitate the high-level design to decide, for instance, the sources of information, where data are processed or how their final consumer uses them. They just deal with the low level details of the network (e.g., cameras or analyzing the images), and lack of suitable abstraction mechanisms. Other approaches [15,31] use general concepts, so the detailed design is too dependent on engineer' skills. For instance, ARTIS [15] and INGENIAS [31] have been used for sensor networks, but their applicability is broader, so their abstractions are necessarily more abstract than ours. When using one of those approaches in the case study, engineers need to decide about issues such as what are agents and what other kind of artefacts in the problem, and what are the specific features that should be added to the standard concepts to model sensors and controllers for fire-fighting. The presented approach extends the INGENIAS metamodel to offer a taxonomy of elements that can appear in these networks, such as resources, controllers and experts, which limits the extent of this issue. Second, previous approaches do not offer specific guidelines about how to carry out the whole development. They usually describe a case study more or less general [30], or a general development process [12,15,31]. Engineers have to decide what information to gather for their models, how their agents should interact to manage the vehicles, exchange information or process data, and which roles are required for that. This approach, through the identification of standard teams in the control, offers an initial way of organizing it that engineers can refine to address the specific requirements of the problem. Moreover, the model-driven development process provides a clear path from the requirements to the code mainly based on automated transformations, reducing the need of manual refinement and coding and reducing unintended mistakes in these tasks.

## 9. Conclusions

The presented approach is intended to facilitate the high-level design of sensor networks based on MAS. It includes an agent-oriented modelling language with specific extensions and an architecture describing how these elements interact to achieve the standard functionalities of these networks.

The modelling language is built around three main concepts. Resources are the passive elements in the network. They are modelled in terms of their available methods. Their sub-types include sensors and data processing utilities. Sensors add to resources a state and work with events, both perceived from their environment and raised to inform to their controllers. These abstractions cover the most common uses of sensors in previous works. The active elements of the network are designed as roles. Roles are common abstraction in MAS defined in terms of their goals, capabilities to achieve them, and their resources and information. Managers are in our approach the roles governing resources. They can have different access rights in order to organize the use of the resources. The final element of the language is teams, which are hierarchical groups of roles aimed at performing some collaborative activities in the network. Actors running in containers implement the roles.

The architecture works with these concepts to specify teams that define standard aspects of behaviour in these networks. It identifies teams for the initialization or redeployment of containers, the management of data (including collection, processing and integration), communications and load balancing (or adaptation of the network to changes in the environment or its elements).

The proposed solution is intended to be flexible in several ways. First, it allows accommodating new or modified concepts for specific needs through changes in its metamodel. Using model-driven techniques, engineers propagate these changes to the supporting tools. Second, the specialization of concepts with inheritance relationships and the organization of systems around teams cover a variety of approaches, so it allows incorporating existing research in the area. Third, the use of a MDE approach facilitates reusing the knowledge present in the definition of teams. These teams can become the basic building blocks for sensor networks with MAS, as their models can incorporate information for the final code generation. For instance, the system in the case study can be largely reused in other settings with dynamic assignment of containers, sensors and tasks. Only models and transformations related with the control of specific sensors and particular manipulations of data need to be replaced in the system. If new teams were required, they could be modelled as extensions of concepts presents in the architecture as done with standard teams.

The main concern in the application of the proposed approach is the difficulty to model the low-level details of sensor networks, such as energy consumption of routing algorithms for messages. At the moment, the only mean to do that is attaching code snippets to entities in design models for the code generation. There are plans to extend the modelling language with additional primitives to describe some low level issues. For instance, methods can be modelled with additional state machines, and certain standard data transformations can be added as instances of the methods of utilities. Moreover, this paper has applied the standard INGENIAS development process for part of its process. Given the particular features of sensor networks, a domain-specific process that considers the kind of artefacts and activities in these developments should be considered. For instance, working with real-time constraints has certain limitations that do not appear in INGENIAS. The already mentioned processes in [12,15,31], combined with our MDE approach, can constitute a suitable starting point for this task.

## Figures and Tables

**Table 1.** Main concepts of the modelling language of the INGENIAS methodology.

**Concept**	**Meaning**
Agent	An active element with explicit goals able to initiate actions involving other elements.
Role	A group of related goals and tasks. An agent playing a role adopts its goals and must be able to perform its tasks.
Environment application	An element of the environment. Agents/roles act on the environment using its actions and receive information from the environment through its events.
Internal application	A non-intentional component of the MAS. Agents/roles use it through its actions and receive information from it through its events.
Goal	An objective of an agent/role. Agents/roles try to satisfy their goals executing tasks. The satisfaction or failure of a goal depends on the presence or absence of some elements (*i.e.*, frame facts and events) in the society or the environment.
Task	A capability of an agent/role. In order to execute a task, certain elements (*i.e*., frame facts and events) must be available. The execution produces/consumes some elements.
Interaction	A basic communication action. Agents/roles send with them information to other agents/roles.
Method	A basic imperative operation of an application described by its parameters and result.
Frame fact	An information element produced by a task, and therefore by the agents/roles.
Event	An information element spontaneously produced by an application.
Interaction	Any kind of social activity involving several agents/roles.
Group	A set of agents/roles that share some common goals and the applications they have access to. The behaviour of groups is specified with workflows involving its components. These workflows indicate their tasks, the elements these produce/consume and the agents/roles that carry them out. The workflows must fulfil the group goals through the achievement of the individual agent/role goals.
Society	A set of agents, roles, applications and groups, along with general rules that govern their behaviour.
Environment	The set of external applications with which the components of a MAS interact.
